# Plasmid-Mediated Fluoroquinolone Resistance Genes in Quinolone-Susceptible *Aeromonas* spp. Phenotypes Isolated From Recreational Surface Freshwater Reservoir

**DOI:** 10.3389/fcimb.2022.885360

**Published:** 2022-05-11

**Authors:** Urszula Kosikowska, Joanna Stec, Sylwia Andrzejczuk, Mariola Mendrycka, Dorota Pietras-Ożga, Dagmara Stępień-Pyśniak

**Affiliations:** ^1^ Department of Pharmaceutical Microbiology, Medical University of Lublin, Lublin, Poland; ^2^ Faculty of Medical Sciences and Health Sciences, Kazimierz Pulaski University of Technology and Humanities in Radom, Radom, Poland; ^3^ Department of Epizootiology and Clinic of Infectious Diseases, Faculty of Veterinary Medicine, University of Life Sciences in Lublin, Lublin, Poland; ^4^ Department of Veterinary Prevention and Avian Diseases, Faculty of Veterinary Medicine, University of Life Sciences in Lublin, Lublin, Poland

**Keywords:** *Aeromonas* spp., surface freshwater, occasional bathing, opportunistic pathogens, PMQR genes

## Abstract

*Aeromonas* spp. are recognized as opportunistic pathogens causing diseases. Infections in humans can result mainly in gastrointestinal and wound diseases with or without progression to septicemia. Although *Aeromonas* spp. are not known uropathogens and they rarely cause urinary tract infection, we hypothesize that the presence of these bacteria in the water and the contact during, *e*.*g*., recreational and bathing activity can create the conditions for the colonization of the human body and may result to diseases in various locations, including the urinary tract. Our study presents the occurrence of aeromonad fluoroquinolone-susceptible phenotypes with the presence of plasmid-mediated fluoroquinolone resistance (PMQR) genes in a natural freshwater reservoir occasionally used for recreational activities. Sixty-nine isolates collected during the bathing period were identified by mass spectrometry and screened for the presence of fluoroquinolone-resistant phenotypes and genotypes. Fluoroquinolone susceptibility was determined as minimal inhibitory concentration values. PMQR *qnr* genes were detected by PCR. Isolates comprising eight species, namely, mainly *Aeromonas veronii* (50.7% isolates) and *Aeromonas media* (24.6% isolates) and rarely *Aeromonas eucrenophila*, *Aeromonas caviae*, *Aeromonas bestiarum*, *Aeromonas ichthiosmia*, and *Aeromonas hydrophila*, were selected. All isolates were phenotypically susceptible either to ciprofloxacin or levofloxacin. Unexpectedly, at least one to three of the PMQR genes were detected in 42.0% of the fluoroquinolone-susceptible *Aeromonas* spp. phenotypes. Mainly the *qnrS* (34.8% isolates) and *qnrA* (14.5% isolates) determinants were detected. In conclusion, the freshwater reservoir occasionally used for bathing was tainted with aeromonads, with a high occurrence of opportunistic pathogens such as *A. veronii* and *A. media*. MALDI‐TOF MS is a powerful technique for aeromonad identification. Our data reveals the mismatch phenomenon between fluoroquinolone-susceptible aeromonad phenotypes and the presence of plasmid-mediated *qnr* resistance genes. It suggests that phenotypically susceptible bacteria might be a potential source for the storage and transmission of these genes. The exposure during, *e*.*g*., a recreational activity may create the potential risk for causing infections, both diagnostically and therapeutically difficult, after expressing the resistance genes and quinolone-resistant strain selection.

## Introduction

The genus *Aeromonas* (family *Aeromonadaceae*) has been described as comprising several species of Gram-negative autochthonic bacteria widely found in different sites in a variety range of habitats. Their principal reservoirs represent the aquatic environment in both surface freshwater and brackish water (Janda and Abbott, 2010). Moreover, *Aeromonas* bacteria were usually found in food products, vegetables, and farm animal fecal contents and as a member of the animal digestive tract microbiota (Janda and Abbott, 2010). However, *Aeromonas* species are commonly described as etiological agents causing animal and human infections (Ghenghesh et al., 2008; Grim et al., 2013; Mosser et al., 2015; Obeidat et al., 2021). These bacteria have been classified into two groups in terms of their host and physiological characteristics: (a) mesophilic (optimal growth temperature range, 35–37°C) and motile aeromonads such as *Aeromonas hydrophila* or *Aeromonas veronii*, which causes different diseases mostly in human and other mammals both in immunocompetent and immunocompromised people—and (b) group of psychrophilic (optimal growth temperature range, 22–25°C) and nonmotile aeromonads such as *Aeromonas salmonicida*, which are the etiological agents of fish diseases (Parker and Shaw, 2011; Dallaire-Dufresne et al., 2014). *Aeromonas* spp. infections are rare and not so important in human health problems; therefore, the pathomechanisms and epidemiology are not very well known. According to some authors, only selected pathotypes of *Aeromonas* spp. with both specific phenotypic and genotypic features can create infections in certain individuals (Grim et al., 2013; Mosser et al., 2015). However, according to earlier studies, aeromonad infections should not be underestimated (Ghenghesh et al., 2008).

The presence of human aeromonad infections was rarely reported in the literature. As opportunistic pathogens, *Aeromonas* spp. are often associated with either animal (*e*.*g*., fish) or human diseases, such as foodborne gastroenteritis and diarrheal illnesses, as well as extraintestinal infections, comprising wound infections with or without progression to septicemia, soft tissue infections, bloodstream infections, meningitis, endocarditis, and osteomyelitis ulcerative disease (Tena et al., 2007; Janda and Abbott, 2010; Alhazmi, 2015; Fewtrell and Kay, 2015; Gauthier et al., 2017; Fernández-Bravo and Figueras, 2020). There are also data on vaginal colonization with *Aeromonas* spp. from healthy pregnant women (Damiain et al., 1995) and patients during labor (Ekwempu et al., 1981). Non-gastrointestinal complications that may arise subsequent to aeromonad infections also include respiratory tract infections and genitourinary or urinary tract infections (UTIs) and hematuria (Bartolomé et al., 1989; Hussain et al., 2018). Most human *Aeromonas* spp. diseases were reported to be associated with mainly three species, including *A. hydrophila*, *A. veronii*, and *Aeromonas caviae*. It has been found to develop in patients with immunocompromised conditions (Chao et al., 2012; Hussain et al., 2018). Moreover, *A. caviae* and *A. hydrophila* were shown as the most common species causing urinary tract infections (Mandal et al., 2010; Chao et al., 2012). *Aeromonas* spp. infections are mostly induced by human activity (*e*.*g*., bathing, swimming, and other recreational activities) in natural reservoirs of surface waters in which these environmental bacteria are widely distributed (Janda and Abbott, 2010; Igbinosa et al., 2012; Fewtrell and Kay, 2015). The number of infection cases increased in the summer months after human contact with *Aeromonas* spp.-contaminated water. Some of these cases were related to a high mortality rate in immunocompromised patients (Bravo et al., 2003; Di Pinto et al., 2012; Igbinosa et al., 2012; Obeidat et al., 2021).

Among others, *Aeromonas* species are able to produce a number of putative virulence factors such as fimbriae, egzotoxins, and hemolysins (Alvandi and Anathan, 2003; Al-Benwan et al., 2007; Chopra et al., 2009; Alperi and Figueras, 2010; Dacanay et al., 2010; Mandal et al., 2010; Dallaire-Dufresne et al., 2014). The importance of bacterial fimbriae as a possible virulence factor in the adhesion process was observed among other known uropathogens such as *Escherichia coli* (Mizunoe and Wai, 1998; Olorunmola et al., 2013). It is widely accepted that fimbriae are the important initiating factors in every UTI through their adhesive properties that allow bacterial adherence to mucous membranes and urinary tract colonization. Fimbriae enable bacteria to survive and multiply *in vivo*. *Aeromonas* species are recognized as opportunistic pathogens causing, among others, UTIs in humans. It is confirmed that the urinary tract is easily accessible to *Aeromonas* spp., and UTIs caused by *A. hydrophila*, *A. veronii* biotype sobria, *Aeromonas popoffii*, and *A. caviae* are scientifically reported as well (Al-Benwan et al., 2007; Dacanay et al., 2010; Janda and Abbott, 2010; Mandal et al., 2010). Waterbathing and other recreational activities within freshwater natural reservoirs may be considered as favorable conditions for exposure to these opportunistic bacteria. When the potential risk associated with external environment and occasional recreation water baths was investigated, attention focused on specific microorganism species and on the ways of their penetration. It is well documented that, due to water contact with human bodies during such recreational activities as swimming, bathing, fishing, canoeing, and other water sports, human infections caused by *Aeromonas* bacteria may occur as a consequence of exposure to these pathogens (Janda and Abbott, 2010; Di Pinto et al., 2012; Igbinosa et al., 2012; Fewtrell and Kay, 2015).

The available literature data show that the pathogenesis and the mechanism of UTIs due to *Aeromonas* spp. have not been explained or described anywhere. Emerging cases of such infections confirm the strong need for attention to these bacteria while investigating for the etiology of UTI, especially in adults with occupational exposure to aquatic ecosystems. Of the patients with such documented diseases, two had a history of occupational exposure to an aquatic environment. The first-ever reported case of UTI infection attributed to *A. popoffii* isolated from freshwater was found in a 13-year-old boy suffering from spina bifida with enterocystoplasty (Hua et al., 2004). Furthermore, *Aeromonas* species were also rarely associated with hemolytic uremic syndrome (Hsueh et al., 1998).

According to Baron et al. (2017), *Aeromonas* spp. are a very good candidates for being indicator bacteria to follow antimicrobial resistance dissemination in aquatic environments. Despite the lack of phenotypically expressed resistance, *Aeromonas* spp. isolates derived from recreational bathing sites may harbor some drug resistance genes or may be the etiological agents of serious opportunistic infections, which is difficult both diagnostically and therapeutically.

For common bacterial infections, including sexually transmitted diseases and urinary tract infections, resistance against a variety of antimicrobials frequently used to treat these infections has been observed worldwide, indicating that we are running out of effective antibiotics [Redgrave et al., 2014]. For UTIs, considered as the most commonly diagnosed diseases in urological patients, fluoroquinolones are regarded as a good option to include in the therapy scheme of UTIs, with good effectiveness and efficacy and a low risk of developing resistant or multi-drug-resistant bacteria (Chao and Farrah, 2019). However, the surveillances demonstrate increasing antimicrobial resistance rates in Gram-negative bacteria, especially *Enterobacteriaceae*, in the past few years. As was reported to the Global Antimicrobial Resistance and Use Surveillance System, the rate of resistance varied from 8.4 to 92.9% for *Escherichia coli* and from 4.1 to 79.4% for *Klebsiella pneumoniae* in reporting countries (WHO, 2021).

Broad-spectrum fluoroquinolones are frequently used to treat UTIs (Parker and Shaw, 2021). These antimicrobials are also very important during a range of *Aeromonas*-infective diseases in human and in animal treatment (Alcaide et al., 2010). The constant persistence of *Aeromonas* in various environments and its increasing resistance are widely observed nowadays (Poirel et al., 2012; Redgrave et al., 2014; Piotrowska and Popowska, 2015; Wimalasena et al., 2017). An aquatic environment creates favorable conditions for the horizontal transfer of resistance genes (Tennstedt et al., 2003). The exposure to resistant bacteria during, *e*.*g*., swimming, bathing, or other activities, may create a potential risk of bacterial influence as opportunistic pathogens. Clinically and environmentally relevant *Aeromonas* spp. are resistant to many agents such as fluoroquinolones on the basis of gene alterations, efflux, and transferable quinolone resistance. Moreover, various clinical and natural water source aeromonads demonstrate greater resistance against different antibiotics (Jacobs and Chenia, 2007; Beaz-Hidalgo and Figueras, 2013). In many cases, *Aeromonas* species resistance relates to the occurrence of mobile resistance genes (Piotrowska and Popowska, 2015; Wimalasena et al., 2017). Although aeromonads are causative uropathogens, the likelihood of their isolation with respect to resistant pathotypes or genotypes from aquatic environments, such as freshwater reservoirs, occasionally used for recreational activity cannot be exaggerated.

The aim of our investigation was to detect the fluoroquinolone-resistant phenotypes and/or genotypes of *Aeromonas* spp. presenting on freshwater surface used only seasonally for recreation and bathing activities. Although *Aeromonas* spp. are not known uropathogens and they rarely cause UTI, we hypothesize that the presence of these bacteria in the freshwater environment, especially with antimicrobial resistance phenotypic and/or genetic factors, and the contact with them during a recreational and bathing activity can create the risk condition for the colonization of the human body and may result to opportunistic diseases in various locations, including the urinary tract. The isolates, obtained from occasional bathing freshwater reservoir, were identified and screened for the following plasmid-mediated quinolone resistance determinants (PMQR): *qnr* (*qnrA*, *qnrD*, and *qnrS*) and *aac-6*′*-Ib-cr*. Hence, it is presumed that these bacteria may pose a risk to the expression of resistance genes under *in vivo* conditions and can cause a difficult-to-treat disease in an infected organism.

## Materials and Methods

### Sample Collection

The samples were collected in natural freshwater reservoir Domaniów (51°26′16.945″ N, 20°50′53.967″ E) which is occasionally used for bathing and other forms of human recreational activities. This freshwater reservoir is also used as a retention tank of Radomka River located in east-central Poland, in the Masovian Voivodeship. The isolates were sampled during bathing season (June 29, 2019). The following sampling sites were chosen based on their location and distance to the beach: I—two places in front of the beach, II and III—two places in the middle of the beach, 1 and 30 m (III) away from the shoreline, respectively, and IV—two places behind the beach. A total of 18 water samples (six sampling locations with three samples taken from each one) were aseptically placed in sterile, dark glass bottles and transported to the laboratory of the Department of Pharmaceutical Microbiology of Medical University of Lublin, Poland. The collected samples were then placed on routinely used microbiological agar media plates (Difco, Detroit, MI, USA) in two different volumes (10 and 100 µl) and in duplicate to differentiate aerobic Gram-negative bacteria. Tryptic soy agar as a nutrient medium for non-selective heterotrophic microorganisms and McConkey agar medium for Gram-negative rods were used to isolate potentially pathogenic bacteria and to distinguish them initially. All plates were incubated at 35°C for 24–48 h. All media were purchased from Difco (Detroit, MI, USA). These culture media were selected to increase the likelihood of isolating microorganisms that are present in a given tank and so to favor the growth of potentially pathogenic bacteria.

### Isolate Identification

An initial phenotypical identification of isolates, according to colonies grown in different morphotypes, was performed. The isolates growing in aerobic conditions (facultative anaerobic bacteria) were previously characterized as Gram-negative, nonlactose-fermenting, and oxidase-positive bacteria. Thus, a total of 71 isolates, growing in various colony morphotypes, were selected from the water samples. These isolates were phenotypically identified at the species level by matrix-assisted laser desorption ionization–time-of-flight mass spectrometry (MALDI-TOF MS) technique using the UltrafleXtreme MALDI-TOF mass spectrometer (Bruker Daltonics, Germany). The classification of *Aeromonas* species based on protein profile detection was described previously (Benagli et al., 2012). The analyses of isolates from surface water samples for *Aeromonas* species were conducted at the Department of Epizootiology and Clinic of Infectious Diseases, Faculty of Veterinary Medicine, University of Life Sciences of Lublin, Poland. The identification was preceded by the extraction of proteins with ethanol and formic acid by using the MALDI-TOF MS technique. Next, sets of bacterial ribosomal proteins were compared with the protein profile reference spectra contained in MALDI Biotyper 3.1 library (Bruker Daltonics, Bremen, Germany). Two major parameters—ion mass-to-charge ratio (*m*/*z*) and relative ion intensity—allow the identification of the bacteria at the genus, species, or strain level. After the protein profile analysis, a total collection of 69 *Aeromonas* spp. isolates identified by MALDI-TOF MS was selected for further analysis. Once the taxonomic position of the microorganism was determined, to determine the relationship between the obtained *Aeromonas* spp. isolates, the MALDI main spectra dendrograms were created.

The proteomic identification step was preceded by a standard ethanol/formic acid extraction procedure, according to the manufacturer’s instructions.

A comparative analysis of collected data with reference bacterial spectra was performed by using MALDI Biotyper 3.1 software (Bruker Daltonics, Germany), comprising 8,468 strains and 47 *Aeromonas* spectra. The report presenting the results included the top 10 identified matches for each sample, along with confidence scores ranging from 0.00 to 3.00. The following score values proposed by the manufacturer were applied: a log (score) <1.70 indicated no reliable identification (-), a log (score) of 1.700–1.999 allowed identification at the genus level (+), a log (score) of 2.00–2.299 indicated highly probable identification at the genus level and probable identification at the species level (++), and a log (score) ≥2.300 indicated highly probable identification at the species level (+++).

### Dendrogram Construction for *Aeromonas* spp. Isolates

To determine the relationship between *Aeromonas* spp. strains, a MALDI main spectra dendrogram was created by using MALDI Biotyper 3.1 software (Bruker Daltonics, Germany). For this purpose, to identify a high level of reproducibility, the spectra were analyzed in FlexAnalysis software and used to create the main spectra profile (MSP). Each MSP was matched against all MSPs of the analyzed set. The list of score values was used to calculate the normalized distance values between strains, resulting in a matrix of matching scores.

### Fluoroquinolone Sensitivity Detection

The antimicrobial susceptibility of 69 *Aeromonas* isolates identified at the species level was determined by VITEK2 Compact Automatic System (bioMerieux, France) using AST-N331 cards containing the following fluoroquinolones: ciprofloxacin and levofloxacin. The bacterial colony suspension equivalent to 0.5 McFarland was diluted in 0.45% saline into 1.5 × 10^7^ CFU/ml, according to the manufacturer’s procedure. The results for *Aeromonas* spp. were interpreted on the basis of minimum inhibitory concentration (MIC) cutoff values according to the European Committee on Antimicrobial Susceptibility Testing (EUCAST) 2021 recommendation and Clinical and Laboratory Standards Institute (CLSI) guideline M45 (CLSI, 2015; CLSI, 2019). On the basis of expert rules, *Pseudomonas aeruginosa* ATCC 27853 (CLSI, 2015; EUCAST, 2021) and *Escherichia coli* ATCC 25922 were used as quality control. Additionally, *A. veronii* DSM 7386 (Deutsche Sammlung von Mikroorganismen, Leibniz-Institut, Germany) was used as positive control.

### DNA Extraction and Fluoroquinolone Resistance Genes

Bacterial DNA was extracted by using the Genomic Mini (A&A Biotechnology, Poland) according to the manufacturer’s protocol. The resulting DNA was stored at 4°C until further analysis. The determination of PMQR genes was performed by PCR amplification from extracted DNA using oligonucleotide primers (Genomed, Poland) with a final concentration of 20 mM (presented in **Table 1**).

**Table 1:** Characteristics of primers used for the amplification of selected fluoroquinolone resistance genes by the PCR method.

Gene	Primer name	Sequence (5′→3′)	Product length (bp)	Reference
*qnrA*	QnrAm-F QnrAm-R	AGAGGATTTCTCACGCCAGG TGCCAGGCACAGATCTTGAC	580	Cattoir et al., 2007
*qnrS*	QnrSm-F QnrSm-R	GCAAGTTCATTGAACAGGGT TCTAAACCGTCGAGTTCGGCG	428	Cavaco et al., 2008
*qnrD*	qnrD-F qnrD-R	CGAGATCAATTTACGGGGAATA AACAAGCTGAAGCGCCTG	582	

The PCR cycling conditions were 34 to 35 cycles of the following: 95°C for 60 s, 50–55°C for 60 s, and 72°C for 60 s. All reactions were carried out using the REDTaq^®^ ReadyMix™ PCR Reaction Mix (Sigma-Aldrich, USA) in a total volume of 25 µl containing 1 µl of each 20 µM primer and 2 µl of extracted DNA, followed by electrophoresis in 1.5% agarose gel (Sigma-Aldrich, USA).

## Results

### Proteomic Identification of Freshwater-Borne *Aeromonas* spp. Isolates Using the MALDI-TOF MS Method

On the basis of protein profile, all 69 (100%) isolates were identified as *Aeromonas* spp. Among these (**Supplementary Table S1**), 67/69 (97.1%) isolates were identified at the species level [log(score) ≥2.0], and two isolates (2.9%) were identified at the level of probable genus identification [log(score) = 1.978–1.99]. A total of eight different *Aeromonas* species were identified (**Table 2**). The most prevalent species were *A. veronii* (50.7%; 35/69 isolates) and *A. media* (24.6%; 17/69 isolates), followed by *A. eurenophila* (7.25%; 5/69 isolates), *A. caviae* (4.35%; 3/69 isolates), *A. ichthiosmia* (4.35%; 3/69 isolates)*, A. bestiarum* (4.35%; 3/69 isolates), *A. hydrophila* (2.9%; 2/69 isolates), and *A. popoffii* (1.5%; 1/69 isolate). These positive identification results were related to members of species presented in the Biotyper database (**Supplementary Table S1**).

**Table 2:** Distribution of *Aeromonas* spp. as the etiological agents of urinary tract infections (UTIs) on the basis of literature data.

Aeromonas species	Number of isolates from recreational water—own data (n = 69)	Number of UTI cases	References
*A. veronii/A. veronii* biovar sobria	35	2	Chao et al., 2012
		1	Hsueh et al., 1998
		2	Mohanty et al., 2020
		2	Mandal et al., 2010
*A. caviae*	3	6	Chao et al., 2012
		4	Mandal et al., 2010
		1	Al-Benwan et al., 2007
*A. hydrophila*	2	12	Chao et al., 2012
		1	Bartolomé et al., 1989
		2	McCracken and Barkley, 1972
		3	Washington, 1972
		1	Mohanty et al., 2020
*A. popoffii*	1	1	Hua et al., 2004

### Dendrogram of the Analyzed *Aeromonas* spp. Isolates

**Figure 1:**
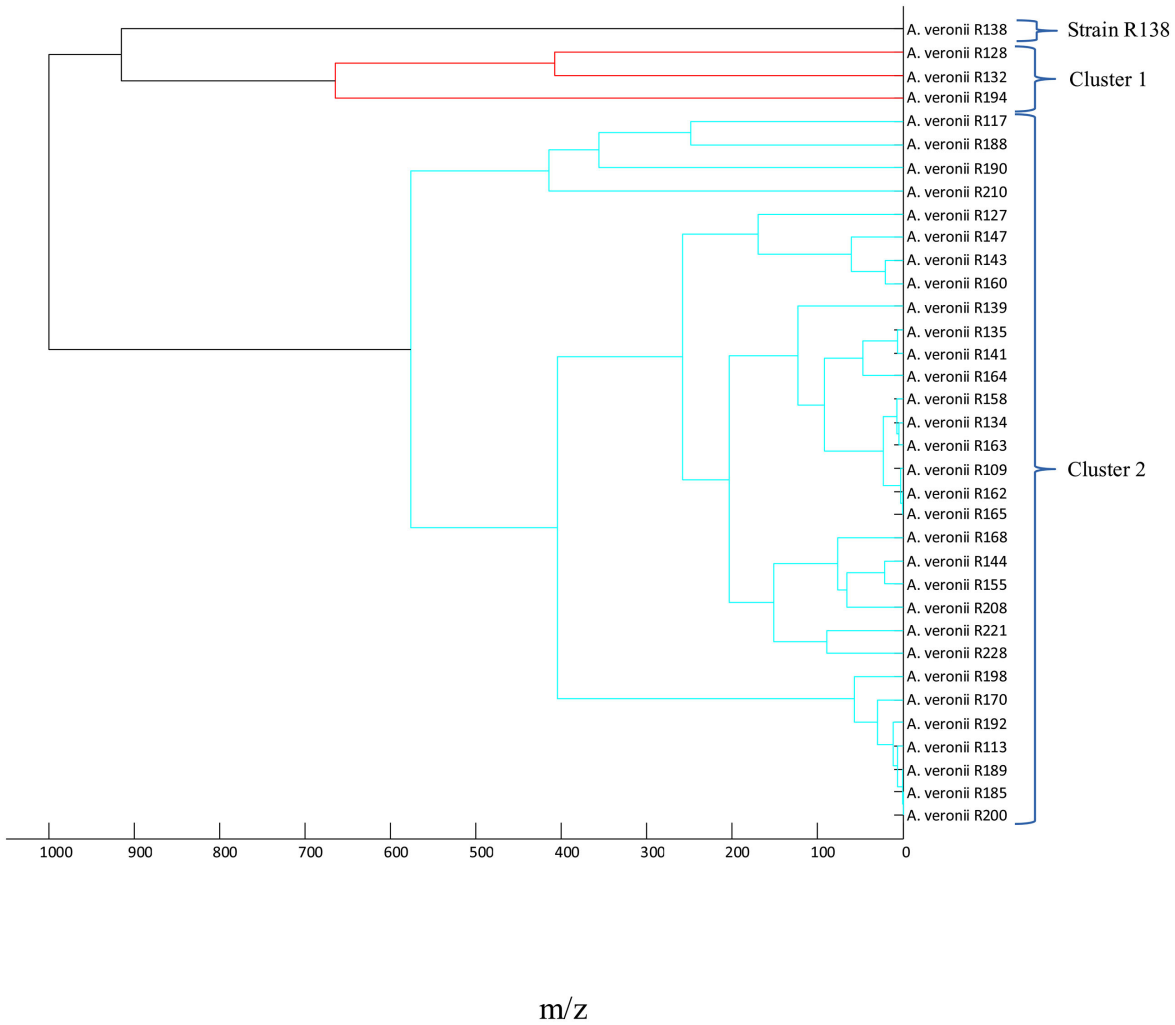
Main spectra profile dendrograms generated by MALDI Biotyper to determine the relationship between *Aeromonas veronii* (*n* = 35) strains.

**Figure 2:**
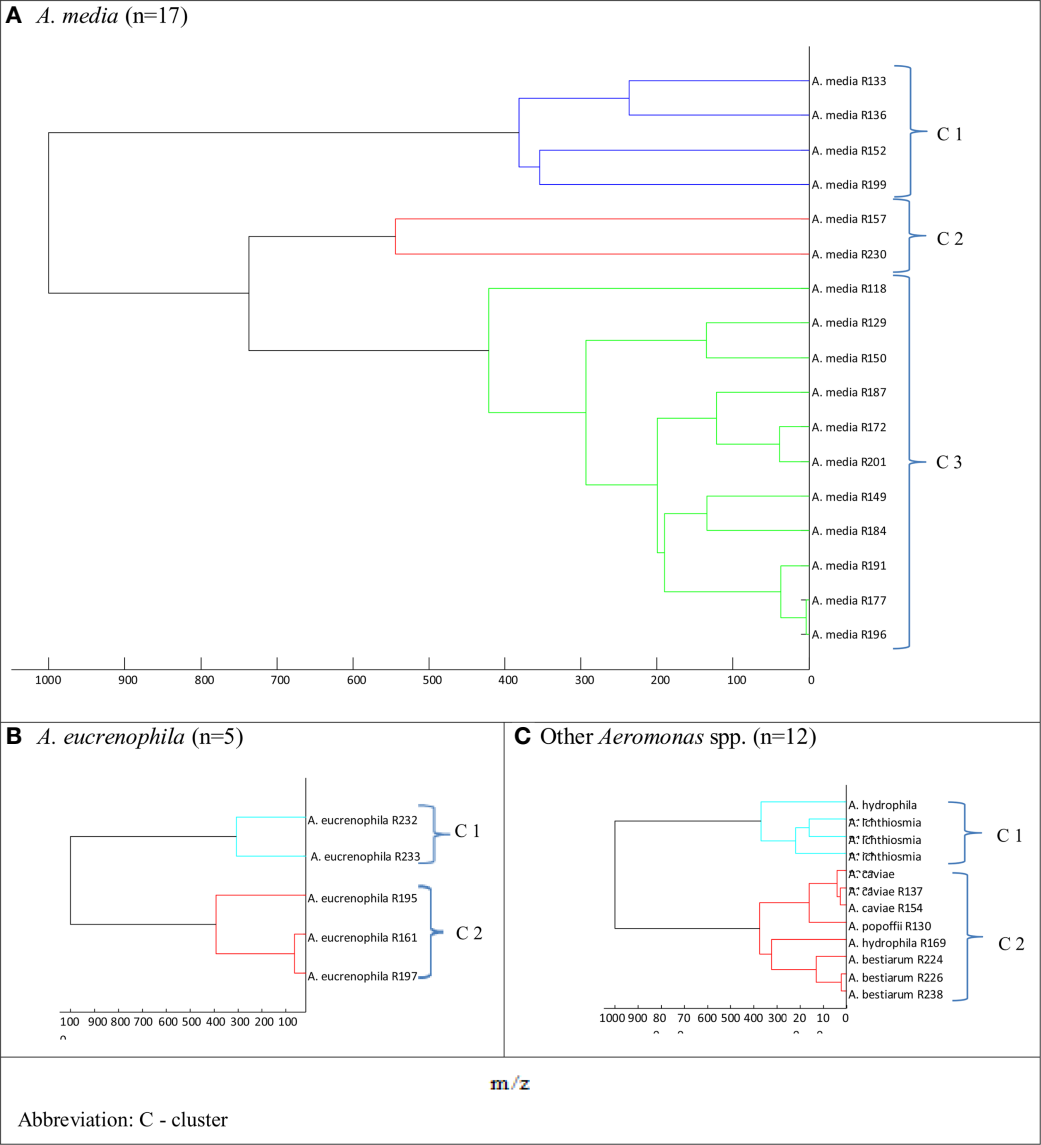
Main spectra profile dendrograms determining the relationship between freshwater reservoir *Aeromonas* strains: **(A)** *Aeromonas media*, **(B)** *Aeromonas eucrenophila*, and **(C)** other *Aeromonas* spp. generated by MALDI Biotyper.

After determination of the taxonomic position of the microorganism, the relationship between identified *Aeromonas* spp. isolates was determined by using the MALDI main spectra dendrograms (**Figure 1** and **Figure 2**). In order to present the results clearly, four dendrograms were prepared for the following species: *A. veronii* (*n* = 35), *A. media* (*n* = 17), *A. eucrenophila* (*n* = 5), and other *Aeromonas* spp. (*n* = 12). First, the *A. veronii* dendrogram was divided into two separate clusters. Cluster 1 included 3 strains with the lowest score value. The largest one, cluster 2 (subclusters 2A and 2B), contained 31 strains, which showed the most closely related strains. The strain R138 remained separately on its own at the distance level between 900 and 1,000 (**Figure 1**).

The dendrograms for *A. media* and rarely identified *Aeromonas* spp. species (*A. hydrophila*, *A. ichtiosmia*, *A. caviae*, *A. popoffi*, and *A. bestiarum*) showed two to three clusters (**Figure 2**). As shown in **Figure 2A**, the dendrogram of *A. media* isolates was divided into 3 more clusters: blue (cluster 1), red (cluster 2), and green (cluster 3) comprising 4, 2, and 11 isolates, respectively. Two other dendrograms, both for *A. eucrenophila* (**Figure 2B**) and other *Aeromonas* spp. (**Figure 2C**), showed branching at the distance level below 400.

### Antimicrobial Susceptibility

**Figure 3:**
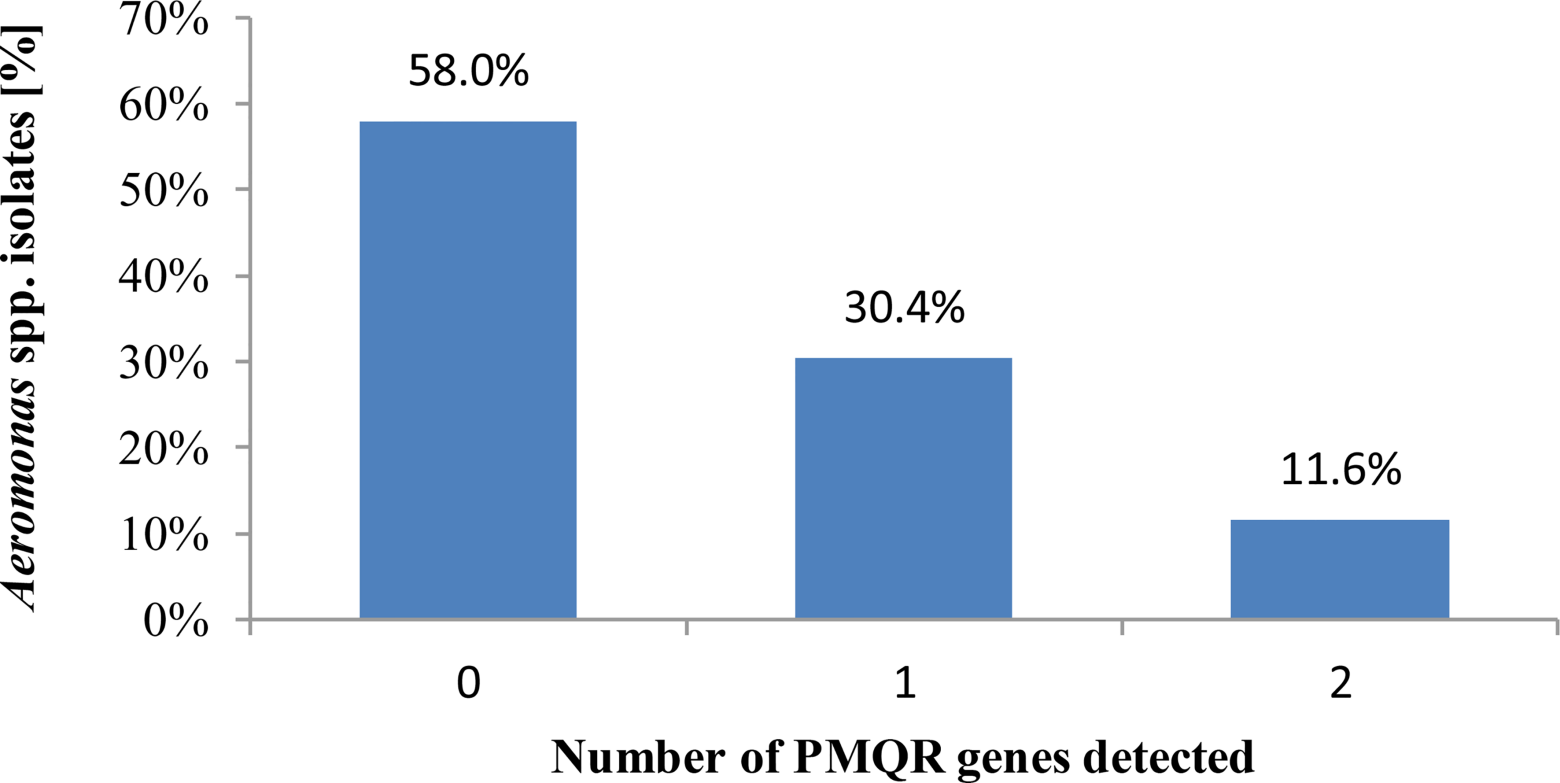
Number of plasmid-mediated fluoroquinolone resistance determinants possessed by *Aeromonas* spp. freshwater-borne isolates.

All *Aeromonas* spp. isolates tested revealed the ciprofloxacin and levofloxacin MIC values of ≤0.25 and <0.5 µg/ml, respectively, so they were categorized as susceptible to these antimicrobials, regardless of the recommendations used to interpret the AST results. Of these, 42.0% (29/69) isolates harbored one or more PMQR genes (**Figure 3**). Co-carriage of two PMQR genes was detected in 11.6% (8/69) isolates.

**Figure 4:**
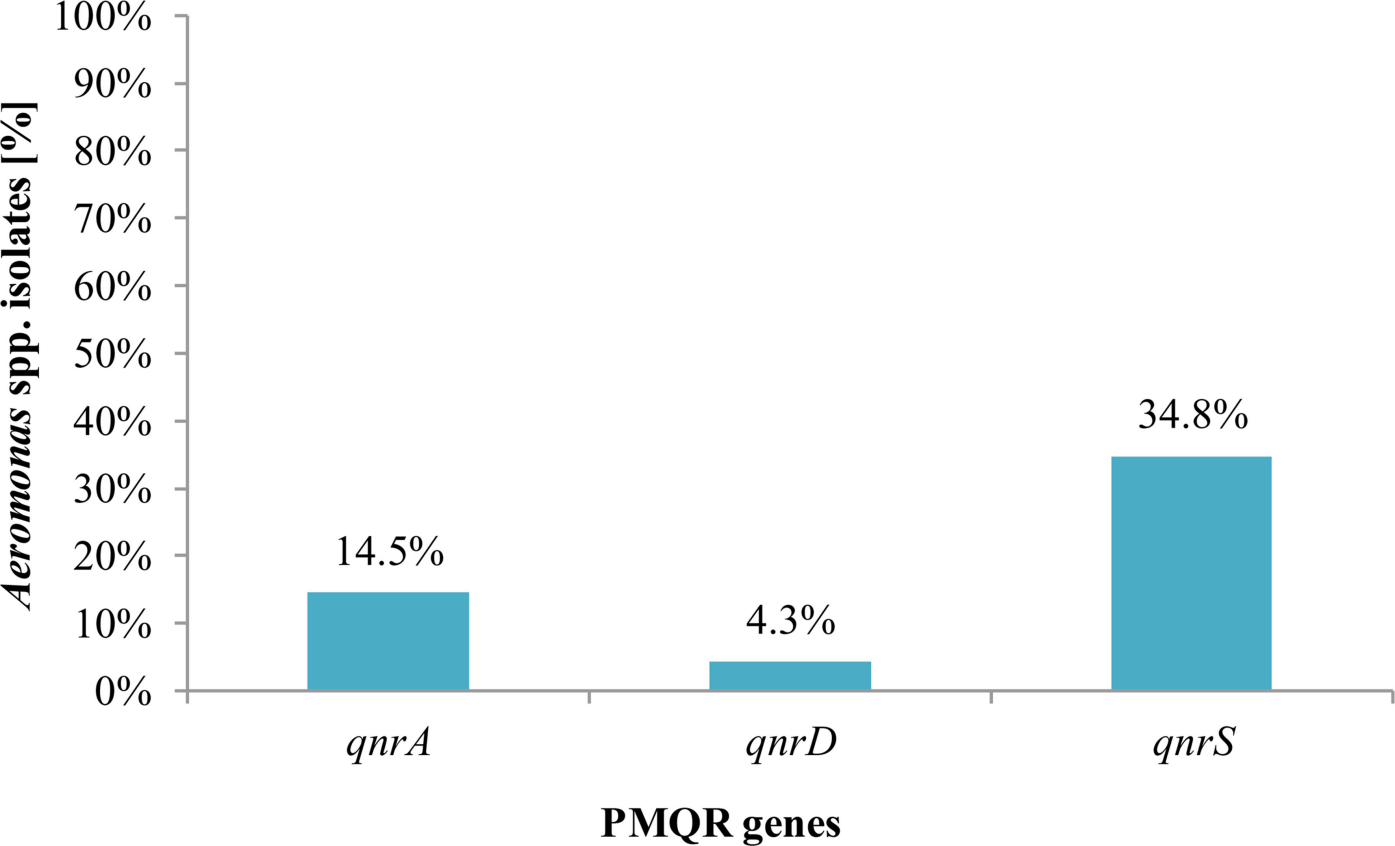
Presence of plasmid-mediated quinolone resistance genes in *Aeromonas* spp. isolates from freshwater samples.

The presence of PMQR genes among *Aeromonas* spp. isolates was confirmed. *QnrS* was the most frequent gene (34.8%, 24/69) of fluoroquinolone-susceptible isolates, which was found in an average of three isolates from eight species tested, followed by *qnrA* (14.5%, 10/69) gene detected in an average of 1.25 isolates from eight *Aeromonas* species (**Figure 4**).

**Figure 5:**
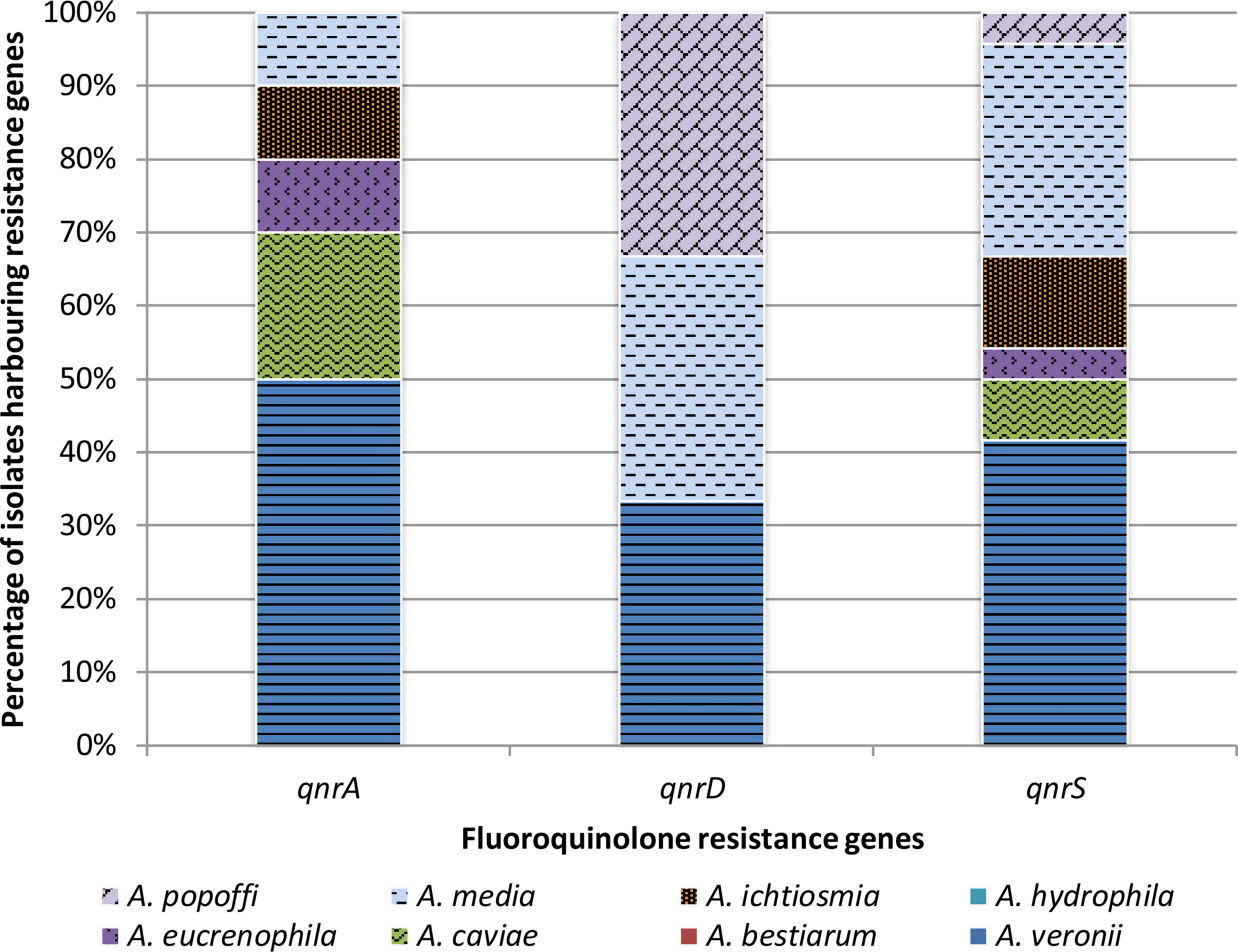
Presence of plasmid-mediated fluoroquinolone resistance genes in different species of *Aeromonas* bacteria isolated from freshwater samples.

**Figure 5** presents the PMQR gene distribution of *Aeromonas* spp. freshwater-borne isolates according to the PCR detection results. Of *Aeromonas* spp., isolates within each of the eight identified species carried at least one PMQR gene, except *A. hydrophila* and *A. bestiarum* isolates. Depending on the species, 23.2% (16/69) *A. veronii* isolates had at least one PMQR gene, while the same factor was in 13.0% (9/69) and 5.8% (4/69) of *A. media* and *A. caviae* isolates, respectively. *QnrD* was the most frequent (10.1%, 7/69) gene among *A. media* isolates, while both *qnrA* and *qnrS* were found in 7.2% (5/69) and 14.5% (10/69) *A. veronii* isolates, respectively.

## Discussion

Given the worldwide distribution of *Aeromonas* genus, the occurrence of virulence factors, and antimicrobial resistance, as well as the ability of these bacteria to survive safety treatments, interest in this genus (especially in its members as human pathogens) has grown over the last years (Sen and Rodgers, 2004; Khajanchi et al., 2010; Pablos et al., 2010). Aeromonad identification, virulence factors, and antimicrobial sensitivity remain poorly understood due to the variable characteristics and behavior of the strains. Furthemore, infective diseases with *Aeromonas* spp. as an etiological agent may be polymicrobial, and they are often difficult to classify.

The environmental microbes found in the natural surface of water reservoir occasionally used for recreational activity may pose a health risk and possibilities of infection by opportunistic pathogens harboring possible resistance against antimicrobial agents. In this work, we studied the presence of *Aeromonas* spp. in the natural reservoir of freshwater occasionally used for bathing and other recreational activities. Additionally, on a large panel of aeromonads, the presence of fluoroquinolone-resistant or fluoroquinolone-susceptible phenotypes of these bacteria was checked automatically using phenotypic methods with the fluoroquinolones such as ciprofloxacin and levofloxacin. Next, the presence of PMQR genes in the examined isolates was observed by the PCR technique. Under favorable conditions, these genes may cause resistance to these drugs important in the treatment of infections, *e*.*g*., in urinary tract diseases. As a consequence of humans’ activity in water, *Aeromonas* spp., as opportunistic pathogens with adhesive properties and possessing virulence factors, may enter the body and colonize it. Then, they may cause diseases, including UTIs, which can be difficult to diagnose and treat.

There are several problems resulting from the widespread presence of *Aeromonas* spp. and their potential to be agents of infections. One is the correct taxonomy and problematic classification (Alvandi and Anathan, 2003; Al-Benwan et al., 2007; Alperi and Figueras, 2010; Mandal et al., 2010), and the other relates to *Aeromonas* spp. drug susceptibility testing and interpretation of its results (Bedearden and Danziger, 2001; Huddleston et al., 2006; Lamy et al., 2012).

In this study, a protein profile based on MALDI-TOF MS technique was used to complete the identification of *Aeromonas* spp. isolated from recreational freshwater. As shown in the literature, the main problem is constantly changing and causing many mistakes in *Aeromonas* spp. taxonomy (Abbott et al., 2003; Beaz-Hidalgo et al., 2010; Vávrová et al., 2015). A key problem in understanding the significance of isolated strains of *Aeromonas* spp. is the choice of the identification method. Traditional microbiological methods (*i*.*e*., morphological, physiological, or biochemical) might not result in the proper identification down to the species level due to the variable characteristics and behaviors of strains (Beaz-Hidalgo et al., 2010). Protein profile-based methods are now becoming more popular and widely accepted in a clinical setting due to their strong reproducibility, simplicity, and high discriminatory power. This technique is used much less frequently in the identification of egzogenic pathogens as well as commensal or symbiotic microorganisms and has limited environmental applicability, whereas most of the currently available mass spectral libraries were developed for human pathogens. It is known that a rapid, cost-effective, and accurate method for the classification of these microbials, such as mass spectrometry (MALDI-TOF MS), would improve our understanding of the microorganisms living in various environments and how to facilitate water use safely. Pinar-Méndez et al. (2021) created a database and defined a MALDI-TOF MS drinking water library. It was developed specifically by targeting bacteria present in drinking or mineral bottled water; however, there is a strong need for such directory for the faster identification of environmental aeromonads isolated from surface freshwater, *e*.*g*., on the basis of our protein profile results, all tested bacteria were identified as *Aeromonas* spp., among which all isolates were described to the species level, including 97.1% ones with log(score) ≥2.0 and only two isolates with log(score) <2.0. During our investigation, mass spectrometry and protein profile-based phenotypic identification were very useful for the classification of the bacteria tested and collected from surface freshwater down to species level.

According to our results, *A. veronii* and *A. media* were the most frequent species among those tested from freshwater-borne isolates. Our data highlighted that *Aeromonas* species present in recreational water should be kept in mind as the probable waterborne opportunistic pathogens important to human health. The mucous membranes in the mouth, nose, as well as respiratory or urinary tract are easily accessible to those bacteria, which may support the hypothesis on *Aeromonas* spp. being the etiological agents of many infective diseases, including as a causal agent in urinary tract infections. *A. hydrophila*, *A. caviae*, and *A. veronii* (biovar *sobria*) are treated as the most common species associated with human infections (Janda and Abbott, 2010; Tang et al., 2014). The cases of UTIs with aeromonad etiology have been proven in the literature, as shown in **Table 2**. As identified, *A. caviae* and *A. hydrophila* were shown as the most common species causing urinary tract infections.

Both the misuse and overuse of medicines used for the prevention and treatment of infections in various organisms, including humans, animals, and plants, appear to be the greatest source of microbial resistance emergence. The importance of various environments’ role in the dissemination of antimicrobial-resistant bacteria is now well recognized. The primary objective of our research on *Aeromonas* spp. isolates from freshwater was to understand the potential risks connected with the exposure to resistant bacteria residing in natural water reservoir seasonally used for bathing and other recreational purposes. It was important to us because of the possible role of various water reservoirs as an ideal place for dissemination and acquisition of antimicrobial resistance by microorganisms forming environmental biomes.

Quinolones are considered to be the most successful and frequently used in many infection therapies, such as diarrhea, skin infections, as well as digestive or urinary system infectious diseases (Pablos et al., 2010; Parker and Shaw, 2011). Hence, we investigated the sensitivity of the tested isolates against 2nd- and 3rd-generation fluoroquinolones, ciprofloxacin and levofloxacin, respectively. An increasing emergence of bacterial resistance and number of various resistance genes detected in *Aeromonas* genus nowadays may be the consequence of antibiotic overuse worldwide. There are numerous genes in the *Aeromonas* spp. genome, antibiotic resistance genes, *e*.*g*., which do not always indicate their phenotypic expression (Grim et al., 2013; Mosser et al., 2015; Chenia, 2016; Wimalasena et al., 2017). Even if a sampled isolate is identified, it is possible that it will obtain the susceptible phenotype in an *in vitro* test. Thus, such bacteria may pose a risk of transmission and expression of resistance genes in *in vivo* conditions and may cause difficulty in the treatment of a disease in an infected organism (Sen and Rodgers, 2004; Rahman et al., 2007). The importance of the drastic upward trend in resistance to quinolones among Gram-negative bacteria is worth noting due also to their wide use in the treatment of infectious diseases during hospitalization as well as in UTI and respiratory tract infections in outpatient settings (Dalhoff, 2012).

Quinolones may inhibit bacterial DNA synthesis by interfering with the action of two crucial enzymes for that process—DNA gyrase and topoisomerase IV (Yoshida et al., 1990; Goñi-Urriza et al., 2002; Soler et al., 2004; Küpfer et al., 2006; Picão et al., 2008; Picão et al., 2013). The clinically important mechanism of bacterial resistance against fluoroquinolones is amino acid substitutions, leading to structural changes in the quinolone resistance-determining regions of DNA gyrase (*gyrA* and *gyrB*) and DNA topoisomerase IV (*parC* and *parE*) subunits, the so-called quinolone resistance-determining regions (QRDR), together leading to target modification. Quinolone resistance may also result from horizontal gene transfer, during which bacteria can acquire various mobile genetic elements, including PMQR genes. It can be mediated by *qnr* genes encoding the pentapeptide repeat family (Küpfer et al., 2006; Grim et al., 2013; Mosser et al., 2015; Chenia, 2016).

Based on the MIC values obtained for a collection of isolates tested, we have shown during our investigation that all *Aeromonas* spp. isolates selected from freshwater were identified as phenotypically susceptible in an *in vitro* test against both ciprofloxacin and levofloxacin. In this study, among the PMQR genes, three *qnr* determinants (*qnrA*, *qnrD*, and *qnrS*) were examined, although during our examination some bacteria were fluoroquinolone resistance silenced gene carriers without their expression. One or more PMQR genes have been reported in the same 42.0% (29/69) of isolates. A very good agreement was observed between the interpretation of quinolone sensitivity results for both CLSI (2015; 2019) and EUCAST [2022] recommendation.

According to Aravena-Roman et al. (2011), the *Aeromonas* spp. strains of environmental origin are not the principial source of resistance. Both antimicrobial resistance mechanisms and its determinants may be acquired from clinical strains. Researchers observed that some common clinical strains, such as *A. veronii* bv. *sorbia* and *A. hydrophila*, were more resistant than the corresponding bacteria isolated from the environment. In contrast, the results of Huddleston et al. (2006) suggested the heavily polluted waters as the source of multiple resistance plasmids. Consistent with these reports, the *in vivo Aeromonas* strains examined during our studies and derived from recreational bathing freshwater created a potential risk as opportunistic pathogens and, after expressing the resistance genes, can cause serious opportunistic infections that are difficult both diagnostically and therapeutically. According to WHO (https://www.who.int/news-room/fact-sheets/detail/antimicrobial-resistance), among the main drivers of resistance against antimicrobials is not only poor infection or disease prevention and control in healthcare facilities and farms but also the lack of awareness and knowledge.

Unfortunately, recommendations and criteria for antimicrobial susceptibility tests and MIC values interpretation of *Aeromonas* spp. were scarce in the guidelines and literature for a long time. *Aeromonas* spp. antimicrobial susceptibility was usually evaluated using *Enterobacteriaceae* breakpoints. According to Lamy et al. (2012), data for *Enterobacteriaceae* ciprofloxacin breakpoints can be accepted for testing *Aeromonas* spp. quinolone sensitivity. Only since 2018 has EUCAST been developing breakpoint tables for the interpretation of MICs and the zone diameters for the genus *Aeromonas* (version 8.0, valid from 2018-01-01). The CLSI M45 document (CLSI, 2015) provided separate limits for *Aeromonas* only, already including in 2015 members of *Aeromonas caviae* complex, *Aeromonas hydrophila* complex, and *Aeromonas veronii* complex.

During our investigations, the PMQR determinant *qnr* (*qnrA*, *qnrD*, and *qnrS*) in the *Aeromonas* spp. isolates obtained from freshwater reservoir has been confirmed in fluoroquinole-susceptible phenotypes. Hence, it is presumed that these bacteria may pose a risk to the expression of resistance genes under *in vivo* conditions and can cause a difficult-to-treat disease in an infected organism, although from the lack of phenotypically expressed quinolone resistance, these bacteria were identified as bearing the susceptible phenotype, which means that, under *in vivo* conditions, these drugs will become ineffective in the infected organism. Over half (58.0%) of the tested *Aeromonas* spp. did not harbor any of the PMQR genes analyzed. Fluoroquinolone usage during infective disease therapy, especially against bacterial infections of the urinary tract, is often the best and cost-effective option between considering the risk and positive effects of treating the patient, which altogether proves their importance. Additionally, it allows to create safe conditions to lower the risk of emerging resistant or multi-resistant pathogens. Among molecular quinolone resistance mechanisms, the most common are mutations both in chromosomal genes encoding gyrase and topoisomerase IV and in regulatory genes which control the expression of efflux pumps present in bacterial membranes. Moreover, among the known three mechanisms of PMQR are Qnr proteins, AAC(6’)-Ib-cr (the aminoglycoside acetylotransferase variant), as well as QepA and OqxAB efflux pumps mediated by plasmids. According to literature, the presence of the two genes simultaneously—*qnrA* and *aac(6’)-lb*—means that the level of resistance for this isolate is increased fourfold more than that conferred by *qnrA* alone (Park et al., 2006; Rodríguez-Martínez et al., 2011; Seyedpour and Eftekhar, 2014). The presence of PMQR genes, such as *qnr* and *aac (6’)-lb-cr*, in *E. coli* fosters a mutation in the QRDR region and the selection of strains resistant to ciprofloxacin and levofloxacin after the use of these drugs in therapy (Park et al., 2006; Piekarska et al., 2015).

The resistance to fluoroquinolones mediated by plasmids is defined to be low-grade resistance with the MIC breakpoint proper for a susceptible strain (Picão et al., 2008; Rodríguez-Martínez et al., 2011; Picão et al., 2013; de Walthoffen, 2020). Moreover, it was detected that the presence of a plasmid in a bacterial cell promotes mutations in the topoisomerase and gyrase genes and the selection of resistance to fluoroquinolones. Genes placed on plasmids may also be localized on other mobile genetic elements, such as transposons and/or integrons, together with genes for resistance to other antimicrobials, like to beta-lactams in strains of multidrug-resistant Gram-negative bacteria. Furthermore, the same plasmids with resistance mechanisms against one antimicrobial may complement other chromosomal resistance types. Natural transformation is the basic way of horizontal gene transfer in microorganisms. Unfortunately, genetic changes naturally occur over time and usually create microorganisms resistant against antimicrobials.

## Conclusions

We confirmed the *Aeromonas* species as a good candidate for bacterial indicators to follow the antimicrobial resistance phenomena and resistance dissemination in aquatic environments. It was shown that the *Aeromonas* genus, being autochthonous in surface freshwater environment, is easy to detect using the proteomic method. We recommend proteomics as a useful method for evaluating species-level freshwater-borne *Aeromonas* identification. The presence of plasmid-mediated fluoroquinolone resistance *qnr* determinants in *Aeromonas* spp. and the higher prevalence of *qnrA* than *qnrS* and *qnrD* was detected in the tested fluoroquinolone-susceptible phenotypes isolated from freshwater. These genes may serve as reservoir for dissemination to other aquatic bacteria and risk of expression *in vivo* in infected humans. Therefore, it is imperative to monitor in *Aeromonas* species the development of antimicrobial resistance to common clinical treatment recommendations, including quinolone susceptibility tests that should be made out. Additionally, the results should be respected in practice for proper and positive results of treatment in water-borne opportunistic infections and to reduce selective pressure that could result in the spread of fluoroquinolone-resistant (uro)pathogens in the environment.

## Data Availability Statement

The datasets presented in this article are not readily available because the given data has been analyzed sufficiently for the purposes of the article. They are not about humans or animals. They are part of a larger project. Requests to access the datasets should be directed to UK (urszula.kosikowska@umlub.pl).

## Author Contributions

UK contributed to the conception and design of the experiment, participated in sampling, and wrote the first draft of the manuscript. UK, SA, and JS organized the database. JS isolated *Aeromonas*, contributed to the design of the study, created tables, and participated in writing of the manuscript. MM participated in sampling and was consulted with the first draft of the manuscript. SA participated in writing the manuscript, wrote the section about molecular method, and created part of the results with gene data; DP-O, and DS-P wrote the methods and results sections of the manuscript, about protein profile detection, and part of data interpretation. All authors contributed to the article and approved the submitted version.

## Conflict of Interest

The authors declare that the research was conducted in the absence of any commercial or financial relationships that could be construed as a potential conflict of interest.

## Publisher’s Note

All claims expressed in this article are solely those of the authors and do not necessarily represent those of their affiliated organizations, or those of the publisher, the editors and the reviewers. Any product that may be evaluated in this article, or claim that may be made by its manufacturer, is not guaranteed or endorsed by the publisher.

## Abbreviations

ATCC, American Type Culture Collection; MIC, minimum inhibitory concentration; CLSI, Clinical and Laboratory Standards Institute; EUCAST, European Committee on Antimicrobial Susceptibility Testing; PMQR, plasmid-mediated quinolone resistance, TSA, triptic soy agar.
