# Supplementary material for: Plasmid-Mediated Fluoroquinolone Resistance Genes in Quinolone-Susceptible Aeromonas spp. Phenotypes Isolated From Recreational Surface Freshwater Reservoir
Source: Front Cell Infect Microbiol. 2022 May 11;12:885360. doi: 10.3389/fcimb.2022.885360 (PMC9132129; doi:10.3389/fcimb.2022.885360)
Supplement: Supplementary file 1 [file Table_1.pdf]

Table 1 suppl

Table 1S. Proteomic identification and diversity of *Aeromonas* spp. isolated from the surface freshwater natural reservoir occasionally used for bathing and other recreational activities during the summer period in Poland

No. of isolate	No. of sample	Species	Score value	Species ID according to MALDI Biotyper 3.1
Place of sampling I - in front of the beach				
**R224**	K17	*A. bestiarum*	2.11	*A. bestiarum CECT 4227T DSM*
**R226**	K19	*A. bestiarum*	2.173	*A. bestiarum CECT 4227T DSM*
**R238**	K31	*A. bestiarum*	2.127	*A. bestiarum CECT 4227T DSM*
**R232**	K25	*A. eucrenophila*	2.106	*A. eucrenophila CECT 4224T DSM*
**R233**	K26	*A. eucrenophila*	2.123	*A. eucrenophila CECT 4224T DSM*
**R223**	K16	*A. ichthiosmia*	2.195	*A. ichthiosmia DSM 6393T HAM*
**R230**	K23	*A. media*	2.202	*A. media DSM 4881T HAM*
**R208**	K1	*A. veronii*	2.177	*A. veronii CECT 5761T DSM*
**R210**	K3	*A. veronii*	2.218	*A. veronii CECT 5761T DSM*
**R221**	K14	*A. veronii*	2.243	*A. veronii CECT 5761T DSM*
**R228**	K21	*A. veronii*	2.247	*A. veronii CECT 4257T DSM*
**R109**	II 1. 3	*A. veronii*	2.172	*A. veronii CECT 5761T DSM*
**R113**	II 1.7	*A. veronii*	2.19	*A. veronii CECT 5761T DSM*
Place of sampling II – 1 m distance from the shoreline beach				
**R131**	II 2.18	*A. caviae*	2.024	*A. caviae CECT 838T DSM*
**R137**	II 2.24	*A. caviae*	2.049	*A. caviae CECT 838T DSM*
**R118**	II 2.4	*A. media*	2.062	*A. media DSM 4881T HAM*
**R129**	II 2.15	*A. media*	2.071	*A. media CECT 4232T DSM*
**R133**	II 2.20	*A. media*	2.094	*A. media DSM 4881T HAM*
**R136**	II 2.23	*A. media*	2.005	*A. media DSM 4881T HAM*
**R130**	II 2.17	*A. popoffii*	2.028	*A. popoffii LMG 17541T HAM*
**R117**	II 2.3	*A. veronii*	2.209	*A. veronii CECT 4257T DSM*
**R127**	II 2.13	*A. veronii*	2.215	*A. veronii CECT 5761T DSM*
**R128**	II 2.14	*A. veronii*	2.041	*A. veronii CECT 4199T DSM*
**R132**	II 2.19	*A. veronii*	2.08	*A. veronii CECT 4199T DSM*
**R134**	II 2.21	*A. veronii*	2.214	*A. veronii CECT 5761T DSM*
**R135**	II 2.22	*A. veronii*	2.183	*A. veronii CECT 5761T DSM*
**R138**	II 2.25	*A. veronii*	2.07	*A. veronii CECT 4199T DSM*
Place of sampling III - 30 m distance from the shoreline beach				
**R154**	II 3.16	*A. caviae*	2.039	*A. caviae CECT 838T DSM*
**R161**	II 3.23	*A. eucrenophila*	2.04	*A. eucrenophila CECT 4224T DSM*
**R156**	II 3.18	*A. hydrophila*	2.005	*A. hydrophila* ssp *hydrophila DSM 30187T DSM*
**R169**	II 3.31	*A. hydrophila*	2.074	*A. hydrophila* ssp *hydrophila DSM 30187T DSM*
**R166**	II 3.28	*A. ichthiosmia*	2.201	*A. ichthiosmia DSM 6393T HAM*
**R167**	II 3.29	*A. ichthiosmia*	2.197	*A. ichthiosmia DSM 6393T HAM*

**R149**	II 3.11	*A. media*	1.978	*A. media DSM 4881T HAM*
**R150**	II 3.12	*A. media*	2.165	*A. media CECT 4232T DSM*
**R152**	II 3.14	*A. media*	2.03	*A. media DSM 4881T HAM*
**R157**	II 3.19	*A. media*	2.143	*A. media CECT 4232T DSM*
**R172**	II 3.34	*A. media*	2.067	*A. media CECT 4232T DSM*
**R139**	II 3.1	*A. veronii*	2.133	*A. veronii CECT 5761T DSM*
**R141**	II 3.3	*A. veronii*	2.241	*A. veronii DSM 17676 HAM*
**R143**	II 3.5	*A. veronii*	2.23	*A. veronii CECT 5761T DSM*
**R144**	II 3.6	*A. veronii*	2.147	*A. veronii DSM 17676 HAM*
**R147**	II 3.9	*A. veronii*	2.114	*A. veronii CECT 5761T DSM*
**R155**	II 3.17	*A. veronii*	2.25	*A. veronii DSM 17676 HAM*
**R158**	II 3.20	*A. veronii*	2.232	*A. veronii CECT 5761T DSM*
**R160**	II 3.22	*A. veronii*	2.152	*A. veronii CECT 5761T DSM*
**R162**	II 3.24	*A. veronii*	2.121	*A. veronii DSM 17676 HAM*
**R163**	II 3.25	*A. veronii*	2.235	*A. veronii DSM 17676 HAM*
**R164**	II 3.26	*A. veronii*	2.137	*A. veronii CECT 5761T DSM*
**R165**	II 3.27	*A. veronii*	2.171	*A. veronii CECT 5761T DSM*
**R168**	II 3.30	*A. veronii*	2.239	*A. veronii DSM 17676 HAM*
**R170**	II 3.32	*A. veronii*	2.246	*A. veronii CECT 4257T DSM*
Place of sampling IV – beyond the beach				
**R195**	T6	*A. eucrenophila*	2.07	*A. eucrenophila CECT 4224T DSM*
**R197**	T8	*A. eucrenophila*	2.093	*A. eucrenophila CECT 4224T DSM*
**R177**	II 4.3	*A. media*	2.087	*A. media DSM 4881T HAM*
**R184**	II 4.10	*A. media*	2.078	*A. media CECT 4232T DSM*
**R187**	II 4.13	*A. media*	2.028	*A. media DSM 4881T HAM*
**R191**	T2	*A. media*	2.038	*A. media DSM 4881T HAM*
**R196**	T7	*A. media*	2.101	*A. media CECT 4232T DSM*
**R199**	T10	*A. media*	2.098	*A. media DSM 4881T HAM*
**R201**	T12	*A. media*	1.99	*A. media DSM 4881T HAM*
**R185**	II 4.11	*A. veronii*	2.204	*A. veronii CECT 4257T DSM*
**R188**	II 4.14	*A. veronii*	2.124	*A. veronii CECT 5761T DSM*
**R189**	II 4.15	*A. veronii*	2.163	*A. veronii CECT 4257T DSM*
**R190**	T1	*A. veronii*	2.104	*A. veronii CECT 4257T DSM*
**R192**	T3	*A. veronii*	2.214	*A. veronii CECT 5761T DSM*
**R194**	T5	*A. veronii*	2.022	*A. veronii CECT 4199T DSM*
**R198**	T9	*A. veronii*	2.237	*A. veronii CECT 5761T DSM*
**R200**	T11	*A. veronii*	2.21	*A. veronii CECT 5761T DSM*
